# MicroRNA-146a constrains multiple parameters of intestinal immunity and increases susceptibility to DSS colitis

**DOI:** 10.18632/oncotarget.5597

**Published:** 2015-09-10

**Authors:** Marah C. Runtsch, Ruozhen Hu, Margaret Alexander, Jared Wallace, Dominique Kagele, Charisse Petersen, John F. Valentine, Noah C. Welker, Mary P. Bronner, Xinjian Chen, Daniel P. Smith, Nadim J. Ajami, Joseph F. Petrosino, June L. Round, Ryan M. O'Connell

**Affiliations:** ^1^ Department of Pathology, University of Utah, Salt Lake City, UT, USA; ^2^ Department of Pathology, University of Utah and ARUP Laboratories, Salt Lake City, UT, USA; ^3^ The Alkek Center for Metagenomics and Microbiome Research, Department of Molecular Virology and Microbiology, Baylor College of Medicine, Houston, TX, USA; ^4^ Department of Medicine, Division of Gastroenterology, University of Utah, Salt Lake City, UT, USA

**Keywords:** miRNA, miR-146a, intestine, microbiota, colitis

## Abstract

Host-microbial interactions within the mammalian intestines must be properly regulated in order to promote host health and limit disease. Because the microbiota provide constant immunological signals to intestinal tissues, a variety of regulatory mechanisms have evolved to ensure proper immune responses to maintain homeostasis. However, many of the genes that comprise these regulatory pathways, including immune-modulating microRNAs (miRNAs), have not yet been identified or studied in the context of intestinal homeostasis. Here, we investigated the role of microRNA-146a (miR-146a) in regulating intestinal immunity and barrier function and found that this miRNA is expressed in a variety of gut tissues in adult mice. By comparing intestinal gene expression in WT and miR-146a−/− mice, we demonstrate that miR-146a represses a subset of gut barrier and inflammatory genes all within a network of immune-related signaling pathways. We also found that miR-146a restricts the expansion of intestinal T cell populations, including Th17, Tregs, and Tfh cells. GC B cells, Tfh ICOS expression, and the production of luminal IgA were also reduced by miR-146a in the gut. Consistent with an enhanced intestinal barrier, we found that miR-146a−/− mice are resistant to DSS-induced colitis, a model of Ulcerative Colitis (UC), and this correlated with elevated colonic miR-146a expression in human UC patients. Taken together, our data describe a role for miR-146a in constraining intestinal barrier function, a process that alters gut homeostasis and enhances at least some forms of intestinal disease in mice.

## INTRODUCTION

The mammalian intestine contains a variety of cell types that coordinate complex processes in order to maintain a barrier between host tissues and resident microbes within the gastrointestinal (GI) tract. An appropriate balance of pro-inflammatory responses to support the barrier, along with mechanisms to ensure the proper degree of immunotolerance, must be achieved to receive the benefits provided by the microbiota while continuing to protect the host from microbial invasion. In order to maintain homeostasis, immunological processes within the gut require proper modulation, as dysregulation has been shown to cause infection, dysbiosis, cancer, and/or autoimmunity [1–3]. Among these unwanted outcomes is inflammatory bowel disease (IBD), which manifests itself as Crohn's Disease (CD) and Ulcerative Colitis (UC) in human patients. IBD affects millions of people around the world, producing a substantial burden on healthcare systems [4]. Identifying risk factors, providing an accurate prognosis, and effectively treating this condition all remain challenging due the complexity of the environmental, genetic, and cellular factors involved in the development of IBD. However, these may be improved as we continue to identify and characterize regulatory mechanisms at play within the gut.

A variety of cell types are involved in promoting intestinal homeostasis, and are made up of both hematopoietic-derived leukocytes and cells of the non-hematopoietic lineage, including intestinal epithelial cells (IECs). Among the immune cells involved, specific T cell lineages play a large role in influencing gut responses. Regulatory T cells (Tregs) are required to maintain tolerance and downregulate gut immune responses [1, 5]; T follicular helper (Tfh) cells interact with B cells in the intestine to help produce antigen-specific antibodies, including IgA, against intestinal microbes to maintain barrier [6–8]; and Th1 and Th17 cells produce specific cytokines to coordinate responses against invading microbes [9, 10]. Th1 cells have been associated with Crohn's Disease in humans, while Th17 cells are implicated both in intestinal tissue damage and in intestinal tissue repair and homeostasis in other contexts [9–12]. The non-hematopoietic derived IECs maintain homeostasis by producing mucus, antimicrobial peptides, and other factors that promote tissue repair, tight junctions, and bacterial targeting [13, 14]. Both T lymphocytes and IECs express cell surface receptors, such as TLRs, that are able to recognize microbial cues and initiate appropriate responses [13, 15]. Downstream from TLRs, signaling through MyD88 and ultimately NF-κB is critical for the proper function of cells within the gut, as disruption of this pathway results in intestinal disease [16–19]. Thus, the proper control of TLR/ NF-κB signaling is essential to overall gut health.

MicroRNAs (miRNAs) are short, non-protein coding RNAs that have been shown to play significant roles in regulating cellular processes within the immune system. miRNAs function to repress their target mRNA genes by binding the 3′-UTR of targets in a mature, RNA-induced silencing complex (RISC)-bound form. Target genes are downregulated approximately 1.5 to 4 fold by miRNAs [20], in such a way that these noncoding RNAs modulate cellular processes in order to maintain equilibrium and/or stability [20–23]. This includes miRNAs within the intestine [24], such as miR-143/145, which have recently been shown to promote IEC regeneration during stress [25]. Among important immune system-related miRNAs is microRNA-146a (miR-146a), which is largely expressed within leukocytes and is induced by pattern recognition receptors and cytokine receptors that activate NFκB [26, 27]. Once induced, miR-146a acts as a negative feedback regulator by targeting TNF receptor-associated factor 6 (Traf6) and IL-1 receptor associated kinase 1 (Irak1), both of which link MyD88 to NFκB signaling [23, 28–30]. miR-146a has also been shown to target Signal Transducer and Activator of Transcription 1 (Stat1) in T cells [31]. Recently, Inducible T-cell costimulator (ICOS), as well as other mRNAs involved in Tfh cell and germinal center biology, were shown to be targets of miR-146a [32]. Through these mechanisms, miR-146a is able to prevent excessive inflammation in response to microbial cues.

Although miR-146a plays a protective anti-inflammatory role within systemic compartments, such as the bone marrow, spleen, lymph nodes and joints [23, 27, 29, 33], its relevance within the adult GI tract remains unclear. This miRNA has been implicated in regulating pathways of intestinal disease [30, 34, 35], although studies with miR-146a−/− mice have not yet been performed in this context. Intestinal immunity and barrier function involve unique cell types and processes that are not found or do not occur in other tissues and are in place to regulate the constant exposure to microbial signals that come from resident intestinal commensal organisms. Thus, we investigated whether miR-146a also plays a host-protective role in this context. We report that miR-146a is expressed in the intestines under the steady state and functions to downregulate a subset of genes and immune cells involved in intestinal barrier function. miR-146a−/− mice displayed an altered microbiota and, surprisingly, were more protected from DSS-induced colitis compared to their WT counterparts. *In vivo* bone marrow reconstitutions demonstrated a contribution by hematopoietic-expressed miR-146a in mediating colitis severity. Consistent with a negative role in intestinal disease, miR-146a was elevated in a cohort of patients with IBD compared to healthy controls. Altogether, through its negative regulation of barrier function, miR-146a limits intestinal health during certain types of stress responses.

## RESULTS

### miR-146a is expressed within the intestines

**Figure 1:**
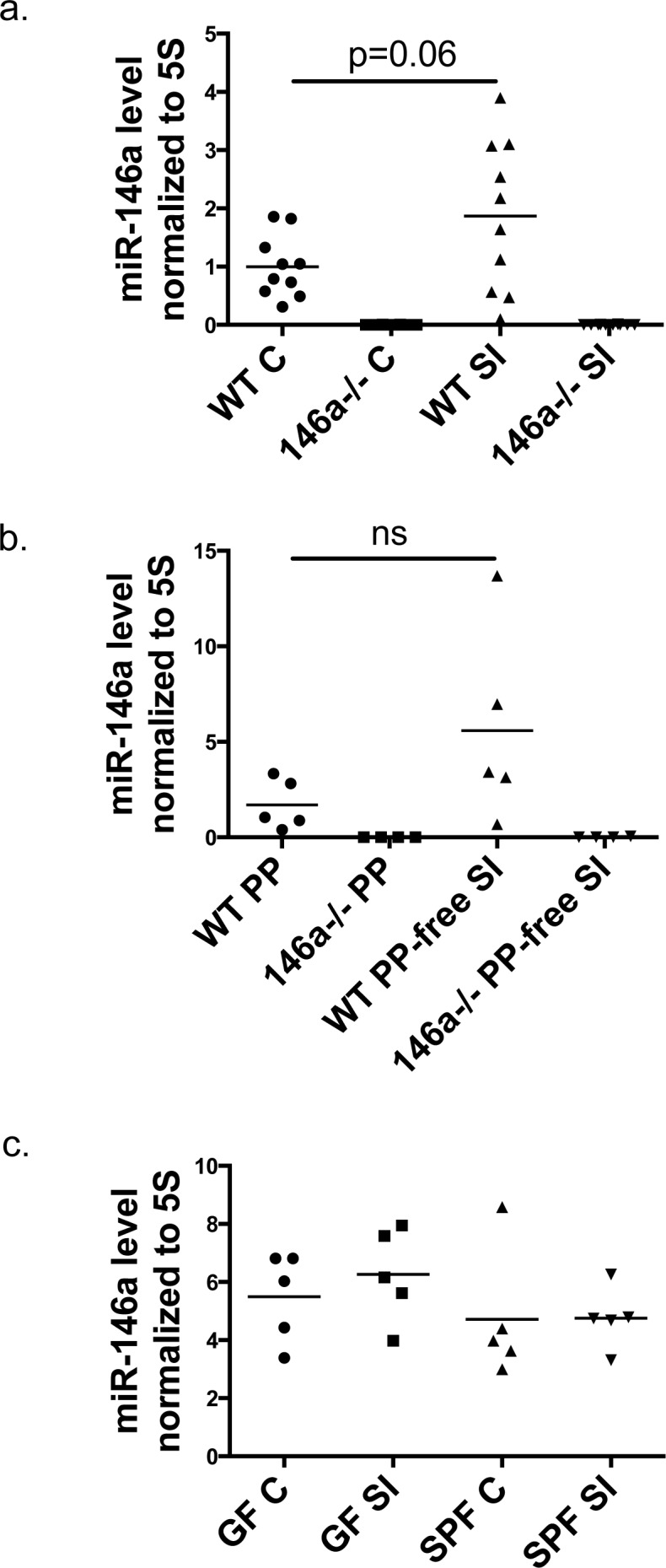
miR-146a is expressed within the intestinal tract Mature miR-146a RNA expression was measured in whole colonic (C) and ileal (SI) tissue of WT C57BL/6 and miR-146a−/− mice via qRT-PCR **a.** RNA was extracted from whole WT and miR-146a−/− Peyer's Patches (PP) and an adjacent piece of small intestinal tissue lacking a PP, followed by qRT-PCR for mature miR-146a expression **b.** Mature miR-146a expression levels were measured in colons (C) and small intestines (SI) of germ-free (GF) and specific pathogen free (SPF) C57BL/6 mice using qRT-PCR **c.** All miR-146a levels were normalized to 5S rRNA; *n* = 10 (a), *n* = 5 (b), *n* = 5 (c).

We characterized the expression profile of miR-146a within gut tissues to begin to identify the cell types in which it may be functioning within the GI tract. miR-146a expression has been well-characterized within hematopoietic cells of the blood, spleen, and bone marrow [36], but its expression within intestinal tissues is not well defined. We found that mature miR-146a is expressed in both the distal colon (C) and in the small intestine (SI) (specifically in the ileum) (Figure 1a). As a control, miR-146a was not detected in intestinal tissues from miR-146a−/− mice. Furthermore, miR-146a expression was compared between small intestinal tissues and adjacent Peyer's Patches (PP), and its levels were similar in both compartments (Figure 1b), indicating that miR-146a levels are not increased in the lymphocyte-rich Peyer's Patch. Because miR-146a has been shown to be induced by TLR/NFκB signaling [26], which can be activated by the intestinal microbiota, we examined mature miR-146a expression in the intestines of germ-free (GF) versus specific pathogen-free (SPF) mice. Equivalent expression within the small intestines and colon was observed when comparing GF and SPF mice, indicating that the presence of the microbiota has little impact on miR-146a levels in the gut (Figure 1c). To determine expression levels of miR-146a in hematopoietic versus nonhematopoietic cells of the intestines, we performed FACS-sorting of CD45^+^ and CD45^−^ cells from mouse colons and small intestines. miR-146a was expressed within CD45^+^ cells of the small intestine and colon, while much lower expression was observed in CD45^−^ cells from these tissues (Supplementary Figure 1a and 1b). Altogether, miR-146a is expressed in a variety of intestinal tissues, primarily within cells of the hematopoietic lineage, and this occurs in a microbiota-independent manner.

### miR-146a represses barrier gene expression in the intestines

**Figure 2:**
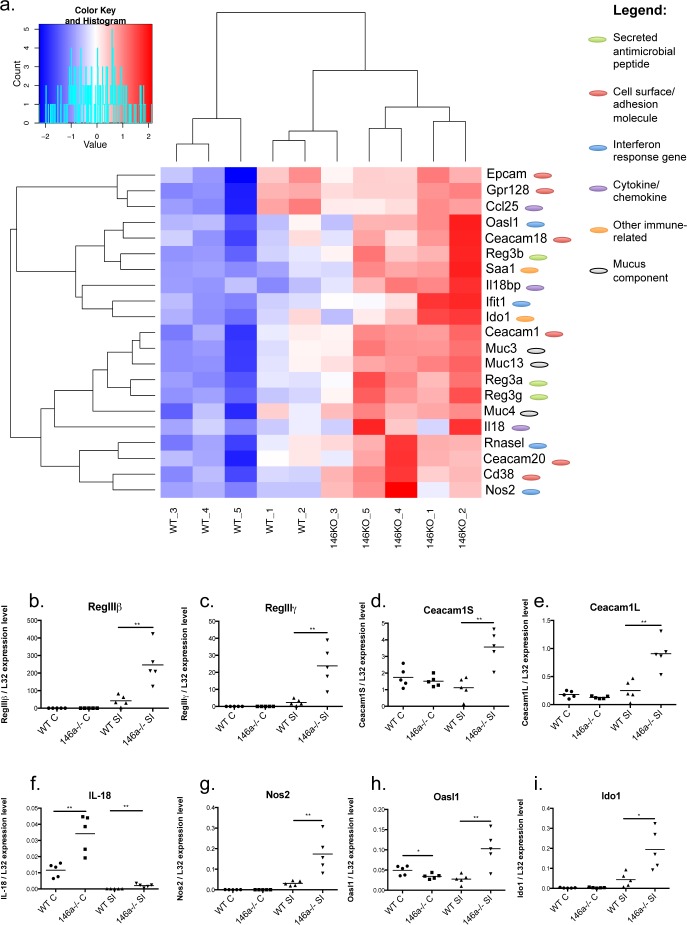
miR-146a regulates expression of genes important for intestinal barrier and homeostasis RNA-seq was performed on a section of ileal tissue of WT C57BL/6 and miR-146a−/− mice. Heat map indicates fold change in gene expression of selected intestinal barrier genes comparing miR-146a−/− mice with WT, *p* < 0.05, Phred-transformed FDR > 10. Genes are categorized by their known functions within the intestine and/or immune system (antimicrobial peptide, cell surface/adhesion, interferon response, cytokine/chemokine, immune-related, and mucus component) **a.** Select genes upregulated in miR-146a−/− small intestines and colons, including Reg3β **b.**, Reg3γ **c.**, Ceacam1S **d.**, Ceacam1L **e.**, IL-18 **f.**, Nos2 **g.**, Oasl1 **h.**, and Ido1 **i.**; were confirmed via qRT-PCR normalized to L32. *n* = 5 (a), *n* = 5 (b-i).

To begin determining the functional role of miR-146a within the intestines, RNA was collected from the distal portions of the colon and ileum of the small intestines of WT and miR-146a−/− mice, and RNA-seq was performed to examine gene expression changes in an unbiased manner. A majority of the significant alterations in gene expression occurred within the small intestine (Supplementary Figure 2a), while substantially fewer differences were seen within the colon (Supplementary Figure 2b). In the small intestine, 289 genes were upregulated and 77 genes were downregulated greater than two fold (FDR > 10) within the small intestines of miR-146a−/− mice compared with equivalent tissues from WT mice (Supplementary Figure 2a). Among the top upregulated genes in miR-146a−/− small intestines were members of the C-type lectin antimicrobial peptide family Reg3: Reg3α, Reg3β, and Reg3γ (Figure 2a), which are expressed by IECs and function to kill gram-positive bacteria [37–39]. Reg3 proteins have been shown to play an essential role in intestinal barrier function and protection from colitis [40], indicating that miR-146a−/− mice have enhanced gut barrier function. Another highly upregulated gene in the miR-146a−/− small intestine was serum amyloid a 1 (*Saa1*), an acute phase, inflammation-promoting gene [41] that has antibacterial effects and is required for protection from colitis [42]. miR-146a−/− small intestines also had higher expression of a number of genes that produce intestinal mucus, including Muc3, Muc4, and Muc13[43]. Other important intestinal barrier genes, including interferon response genes RNaseL, Oasl1, Nos2 and Ifit1 [44–46], as well as intestinal cell adhesion molecules Ceacam1, Ceacam20, Ceacam18, and Epcam, were expressed at higher levels in miR-146a−/− small intestines compared to WT [47–49]. Futhermore, a subset of genes that make up epithelial cell junctions and enhance the intestinal barrier, including claudins, occludin, and e-Cadherin [50], were modestly upregulated in mice lacking miR-146a (Supplementary Figure 3b). Also of note, the cytokine IL-18 and regulator protein IL18bp, in addition to the enzyme Ido1, were at higher levels in the absence of miR-146a. Increased IL-18 [51, 52] and Ido1 [53] levels have been shown to play a protective role in mice during experimental colitis. Several of these gene expression changes within the small intestine were confirmed via qRT-PCR including Reg3β, Reg3γ, Ceacam1 (short and long isoforms), IL-18, Nos2, Oasl1, and Ido1 (Figure 2b–2i). Also of interest, IL-18 expression was found to be significantly upregulated in the colons of miR-146a−/− versus WT mice (Figure 2f). Using Targetscan and miRTarBase, 18 predicted and confirmed miR-146a targets were found to be derepressed within whole ileal tissues of miR-146a−/− mice compared with WT controls (Supplementary Figure 3a). Derepression of the confirmed miR-146a target NUMB correlated with decreased expression of downstream Shh signaling genes (Supplementary Figure 3c), which can play a role in IBD pathology [35]. Altogether, these data demonstrate that miR-146a functions to repress genes involved in intestinal barrier function and immunoprotective inflammation.

In order to identify signaling pathways that miR-146a may be directly regulating within the small intestine, we performed a pathway analysis of the RNA-seq data using the IPA software program from Ingenuity. Among the top upstream regulators predicted to be activated in the absence of miR-146a were the cytokines IL-18 and IL-22; the IL-1R, IL-18R and TLR adaptor protein MyD88; the transcription factors NFκB (RelA), Stat1, and Stat3; and type I and II interferons IFNβ and IFNγ (Supplementary Figure 4a). This predicted enhancement in signaling by MyD88 and NF-κB, as well as enhanced interferon and Stat1 signaling, is consistent with previous data demonstrating that miR-146a can directly repress Traf6 and Irak1 (both downstream from MyD88 and upstream of NFκB) [26, 27, 29] as well as Stat1 (IFN pathway) [31]. Further, Ingenuity predicted that miR-146a−/− mice should be resistant to inflammatory bowel disease (IBD) and colitis (Supplementary Figure 4b). Based upon these results and on previous findings that MyD88 [16, 18, 38, 54] and downstream signals promote intestinal barrier function, our data suggest that miR-146a−/− mice have enhanced barrier function within their intestinal tract through a mechanism involving enhanced MyD88 signaling.

### miR-146a constrains Th17 and Treg populations within the lamina propria

**Figure 3:**
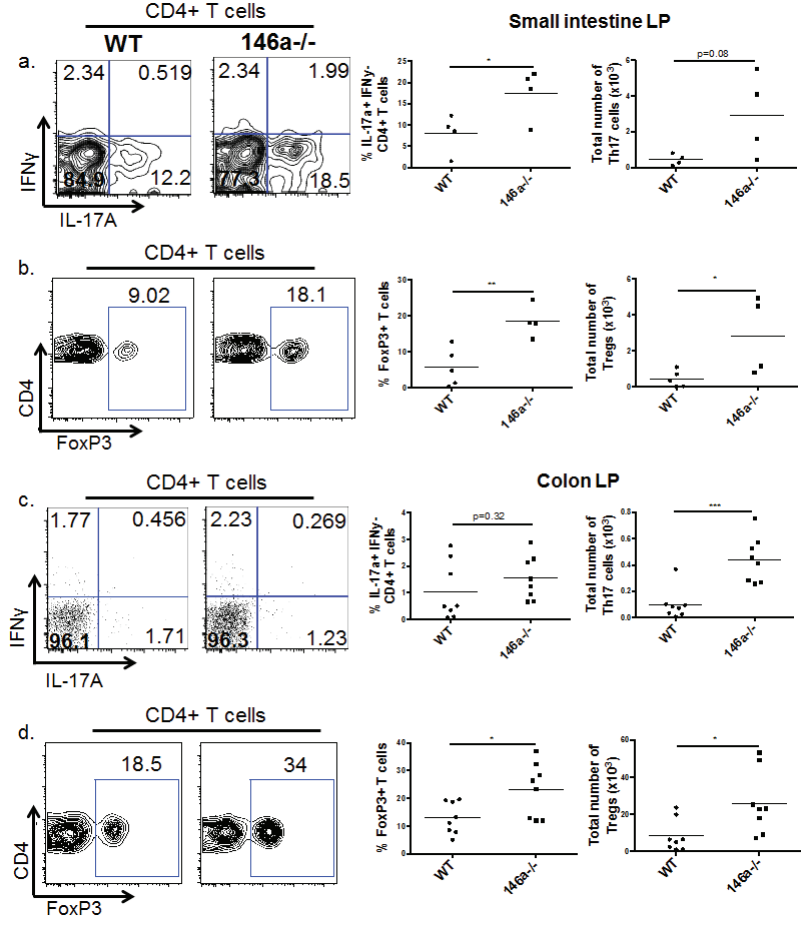
miR-146a constrains Th17 and Treg populations in the lamina propria Lymphocytes were isolated from the small intestinal lamina propria, and flow cytometry was utilized to examine immune cell populations within this tissue. Displayed are representative flow plots (Wt on the left and miR-146a−/− on the right), percentages, and total numbers of IL-17A^+^ IFNγ^−^ (Th17) CD4^+^ CD3e^+^ T cells within the small intestine LP. All populations were first gated on lymphocytes using the FSC/SSC gate, then on CD3e^+^ CD4^+^ cells, followed by the IL-17A and IFNγ gating shown **a.** Representative flow plots, percentages, and total numbers of FoxP3^+^ (Treg) CD4^+^ CD3e^+^ T cells within the small intestine LP. All populations were first gated on lymphocytes using the FSC/SSC gate, then on CD3e^+^ CD4^+^ cells, followed by the FoxP3^+^ gating shown **b.** Displayed are representative flow plots, percentages, and total numbers of IL-17A^+^ IFNγ^−^ (Th17) CD4^+^ CD3e^+^ T cells within the colonic LP. All populations were first gated on lymphocytes using the FSC/SSC gate, then on CD3e^+^ CD4^+^ cells, followed by the IL-17A and IFNγ gating shown **c.** Representative flow plots, percentages, and total numbers of FoxP3^+^ (Treg) CD4^+^ CD3e^+^ T cells within the colonic LP. All populations were first gated on lymphocytes using the FSC/SSC gate, then on CD3e^+^ CD4^+^ cells, followed by the FoxP3^+^ gating shown **d.** *n* = 5 (WT) and 4 (miR-146a−/−) (a, b), *n* = 8 (c, d).

In addition to the barrier gene products described above, leukocytes are also critical regulators of intestinal barrier function and participate in the regulation of these genes. Thus, we examined immune cell populations of the small intestinal and colonic lamina propria (LP), comparing WT to miR-146a−/− mice. Few differences were observed in LP myeloid (CD11b^+^) and B cell (B220^+^) proportions and numbers when comparing WT and miR-146a−/− mice (Supplementary Figure 5b, 5c, 5e, and 5f). However, miR-146a−/− mice had an expansion of CD4^+^ T cells within the small intestinal and colonic LP (Supplementary Figure 5a and 5d). Upon examination of specific CD4^+^ helper T cell populations, we observed an increase in Th17 and Treg cells in miR-146a−/− mice, both in small intestinal (Figure 3a and 3b) and colonic (Figure 3c and 3d) LP. Altogether, these results demonstrate that miR-146a plays a role in restricting CD4^+^ helper T cell populations within the intestinal lamina propria, primarily of the Th17 and Treg cell subsets. These cell subsets have previously reported functions in intestinal barrier and tolerance [5, 11, 12], including the regulation of several barrier genes, indicating that miR-146a is playing an immunomodulatory role to shape the intestinal immune landscape.

### miR-146a constrains germinal center reactions in the Peyer's Patch

**Figure 4:**
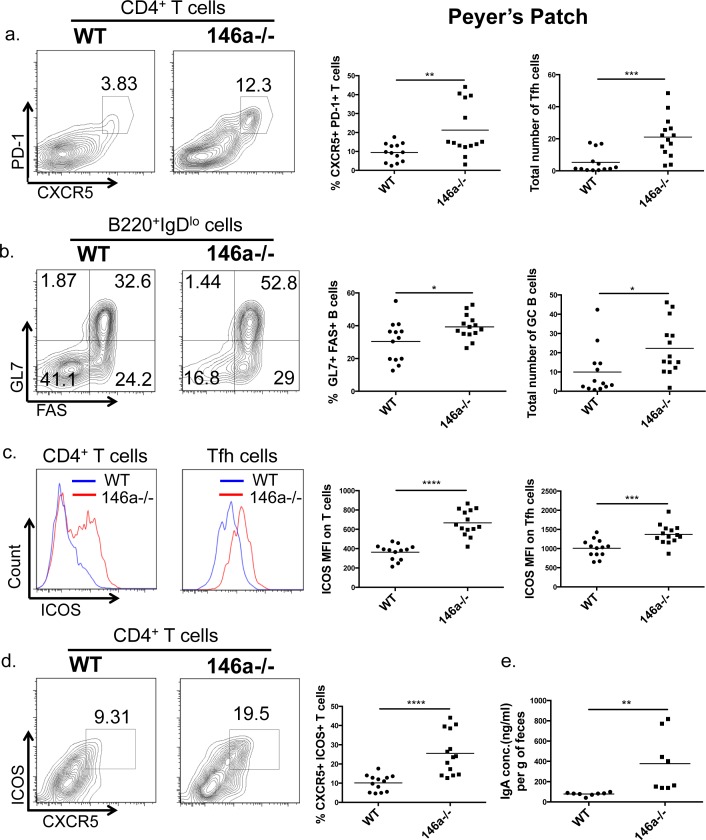
miR-146a constrains Tfh, germinal center (GC) B cell, and IgA levels in the intestine Peyer's Patches were collected from WT C57BL/6 and miR-146a−/− mice, and flow cytometry was utilized to examine T and B cell populations within these GALT structures. Displayed are representative flow plots, percentages, and total numbers of CXCR5^+^ PD-1^+^ (Tfh) CD4^+^ CD3e^+^ T cells within the PP. All populations were first gated on lymphocytes using the FSC/SSC gate, then on CD3e^+^ CD4^+^ cells, followed by the PD-1 and CXCR5 gating shown **a.** Representative flow plots, percentages, and total numbers of GL7^+^ FAS^+^ (GC B) B220^+^ IgD^lo^ B cells within the PP are shown. All populations were first gated on lymphocytes using the FSC/SSC gate, then on B220^+^ IgD^lo^ cells, followed by the GL7 and FAS gating shown **b.** Representative flow plots and quantification of mean fluorescence intensity (MFI) of ICOS on CD3e^+^ CD4^+^ T cells (left histogram) and Tfh cells (right histogram), measured via flow cytometry with a fluorescent antibody against ICOS. All populations were first gated on lymphocytes using the FSC/SSC gate, then on CD3e^+^ CD4^+^ cells, followed by PD-1^+^ and CXCR5^+^ for Tfh cells **c.** Shown are representative flow plots and percentages of CXCR5^+^ ICOS^+^ CD4^+^ CD3e^+^ T cells within the PP. All populations were first gated on lymphocytes using the FSC/SSC gate, then on CD3e^+^ CD4^+^ cells, followed by the ICOS and CXCR5 gating shown **d.** Feces pellets were collected from WT and miR-146a−/− mice, weighed, and homogenized; total IgA was measured in fecal homogenates via ELISA **e.** *n* = 13 (WT) and 14 (miR-146a−/−) (a-d), *n* = 8 **e.**

In addition to the LP, T lymphocytes of gut-associated lymphoid tissues such as Peyer's Patches (PP) are also critical regulators of intestinal barrier function. In particular, T follicular helper (Tfh) cells and their interactions with germinal center (GC) B cells within the intestinal PP are essential to produce IgA and maintain intestinal homeostasis [55]. Because miR-146a plays a role in Tfh and GC B cell accumulation in extra-intestinal tissues [32, 56] and is expressed within the PP (Figure 1b) we examined lymphocyte populations within this gut-associated lymphoid tissue. miR-146a−/− mice displayed an expansion in both the percentage and total number of PD-1^+^ CXCR5^+^ Tfh cells within the PP compared with WT mice (Figure 4a). Tfh cells interact with GC B cells within the PP to promote antigen-specific IgA responses [57]. Accordingly, the observed expansion of Tfh cells corresponded with an increase in GL7^+^ FAS^+^ GC B cells in the PP (Figure 4b). Inducible costimulator (ICOS) expression on Tfh cells is essential for their differentiation and function during germinal center responses [58] and is a direct target of miR-146a [32]. Within the PP, we observed a significant increase in ICOS expression (MFI) on total CD4^+^ T cells and on Tfh cells upon loss of miR-146a (Figure 4c), consistent with elevated Tfh, GC B cells, and a previously reported role of miR-146a in Tfh function [32]. Furthermore, the percentage of CXCR5^+^ Tfh cells expressing ICOS was increased in miR-146a−/− mice compared with WT (Figure 4d). Consistent with an enhanced germinal center reaction, total fecal IgA levels were also elevated in miR-146a−/− compared with WT mice (Figure 4e). Tfh cells, GC B cells, and IgA have all been shown to play an important role in promoting intestinal barrier and in shaping microbial populations within the gut [55, 59–61]. Altogether, these results indicate that miR-146a−/− mice have increased Tfh and GC B cells within the intestine, correlating with elevated T cell ICOS expression and IgA levels.

### miR-146a−/− mice are resistant to DSS-induced colitis

**Figure 5:**
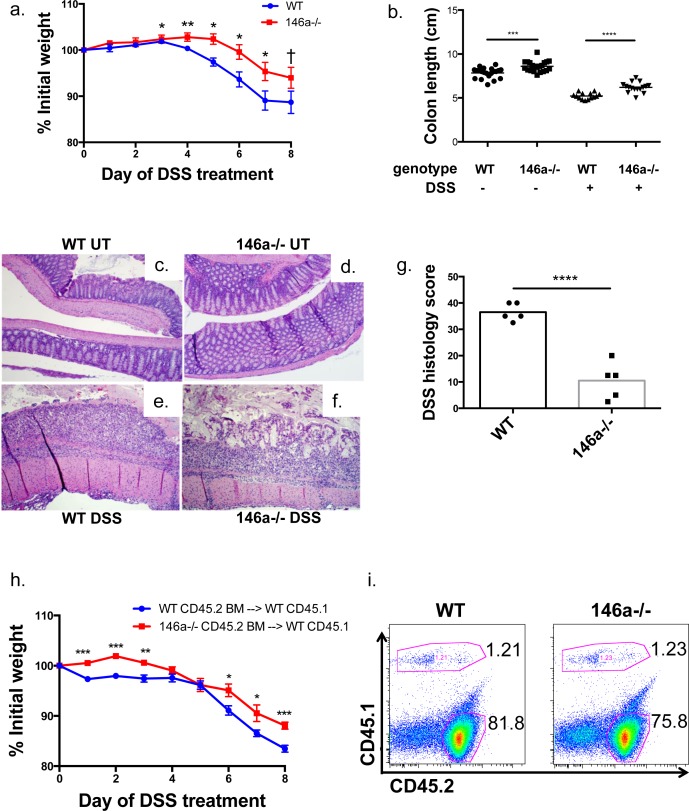
miR-146a−/− mice are protected from DSS-induced colitis WT C57BL/6 and miR-146a−/− mice were treated with 3.5% dextran sodium sulfate (DSS) for eight days. Weight change of WT and miR-146a−/− mice during DSS colitis, as measured by percent initial weight **a.** Colon lengths of WT and miR-146a−/− mice before (left columns) and after DSS treatment (right columns) **b.** H&E staining of untreated (UT) and DSS-treated WT and miR-146a−/− colonic sections, shown at 10x magnification **c.**-**f.** Histology scores of H&E stained DSS-treated colon tissues, based on grade 3 and 4 mucosa loss, as scored by a blinded pathologist **g.** Weight change during DSS colitis following bone marrow transfer of WT or miR-146a−/− CD45.2 bone marrow into lethally irradiated WT CD45.1 recipients **h.** Representative flow cytometry plots showing percentages of donor (WT or miR-146a−/− CD45.2^+^) and recipient (WT CD45.1^+^) splenocytes following bone marrow reconstitution **i.** *n* = 16 (a-b); *n* = 5 (c-g); *n* = 15 (h-i).

Altered intestinal inflammatory responses and barrier function have been linked to diseases within the intestinal tract. Because our RNA-sequencing (Supplementary Figure 4a and 4b) and immunological data suggested that miR-146a−/− mice might be resistant to inflammatory bowel disease (IBD) and colitis, we utilized the dextran sodium sulfate (DSS) murine colitis model to examine barrier function and disease susceptibility within the intestines. This model of intestinal inflammation, which affects both the colon and small intestine, models human Ulcerative Colitis [62–64]. After 8 days of DSS treatment, WT mice lost significantly more weight than miR-146a−/− mice (Figure 5a), and miR-146a−/− colons were longer in both DSS-treated and untreated mice (Figure 5b). The net change in colon length following DSS administration was less in miR-146a−/− mice compared to controls (Supplementary Figure 6a). Upon examination of H&E stained colonic sections by a blinded pathologist, WT colons had a complete loss of crypts and intestinal architecture, and a large infiltration of leukocytes (Figure 5e). DSS-treated miR-146a−/− colons appeared diseased, but some crypts were still in tact and inflammation and tissue damage were reduced (Figure 5f). Both WT and miR-146a−/− colons that were untreated appeared normal (Figure 5c and 5d). Altogether, WT mice showed higher histological colitis scores compared with mice lacking miR-146a (Figure 5g). Decreased expression of TNFα and IL-6 mRNAs within colons of miR-146a−/− DSS-treated animals also indicated reduced disease severity (Supplementary Figure 6b and 6c).

In order to understand the contribution of miR-146a within hematopoietic versus non-hematopoietic cells to DSS colitis susceptibility, we performed bone marrow reconstitutions, where WT or miR-146a−/− CD45.2^+^ bone marrow was transferred to lethally irradiated WT mice carrying the CD45.1 marker. Following hematopoietic cell reconstitution, DSS was administered to bone marrow recipient animals. WT mice that received miR-146a−/− bone marrow were again more protected from DSS colitis compared with WT bone marrow recipients, as shown by weight loss differences (Figure 5h). Flow cytometric analysis of the spleen revealed that recipient bone marrow (CD45.2^+^) made up a majority of the hematopoietic cells, indicating that the reconstitution was effective (Figure 5i). These data demonstrate that the observed colitis phenotype is mediated by miR-146a within the hematopoietic compartment. Altogether, our data reveal that miR-146a decreases intestinal barrier function resulting in elevated DSS colitis severity.

Upon examination of the intestinal landscape, one cannot ignore the crosstalk between host tissues and the 10^14^ commensal organisms that reside in this locale [65]. Owing to the clear differences in intestinal barrier and immunological parameters between WT and miR-146a−/− mice, we hypothesized that alterations to the microbiota must be taking place. To directly test this, we performed Illumina sequencing of 16s rDNA extracted from fecal pellets of both genotypes to determine if there were differences in microbial populations. Both unweighted and weighted, normalized Unifrac distances demonstrated significant differences between total microbial communities in WT versus miR-146a−/− mice (Supplementary Figure 7a). Larger and more significant Unifrac distances were observed using the unweighted measure, which is more dependent upon differences in rare bacterial taxa. Alpha diversity, as measured by multiple indices, was not significantly different between WT and miR-146a−/− mice, although a larger distribution in alpha diversity was observed in the knockout group (Supplementary Figure 7b). Upon comparison of microbial taxa that varied between WT and miR-146a−/− mice, statistically significant shifts at the phyla level were not observed (Supplementary Figure 7c). Altogether, these data indicate that host miR-146a plays some role in shaping the gut microbiota by regulating the magnitude of host-barrier function, albeit commensal community shifts were minor and within rare taxonomic groups.

Because miR-146a−/− mice had some alterations in gut microbiota composition compared to WT controls, we tested the direct contribution of these small community shifts to disease susceptibility. WT or miR-146a−/− mouse gut microbiota was transferred to broad-spectrum antibiotic pre-treated WT recipient mice via oral gavage, and DSS colitis was induced following transfer. We observed no difference in DSS disease severity when comparing recipients having received WT or miR-146a−/− microbiota. This provides evidence that the microbiota does not directly facilitate the phenotypic differences in colitis symptoms (Supplementary Figure 7d). Altogether, our data reveal that miR-146a decreases intestinal barrier function resulting in elevated DSS colitis severity, and this involves hematopoietic cells and appears to be microbiota-independent.

### miR-146a is overexpressed in patients with ulcerative colitis

**Figure 6:**
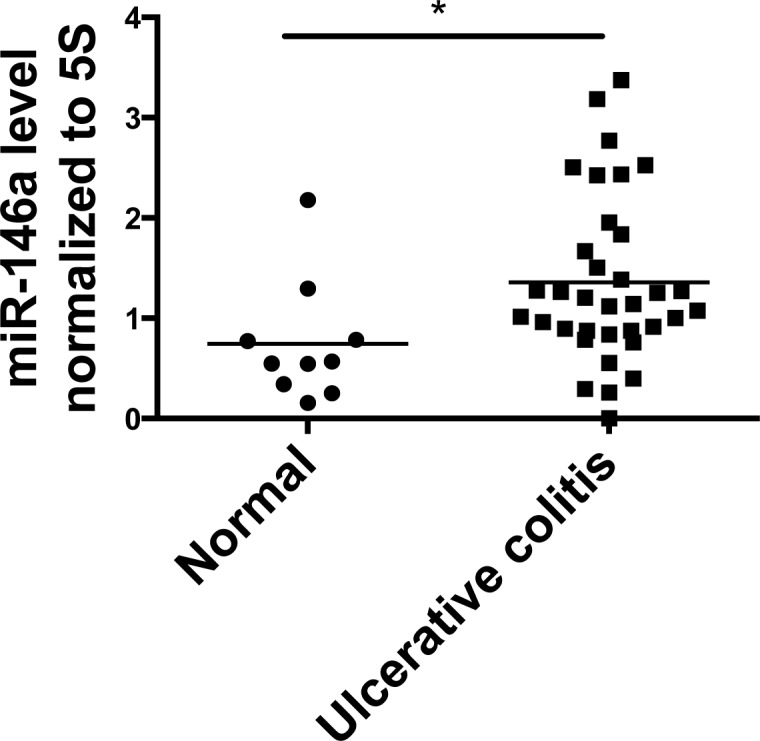
miR-146a expression is elevated in colon tissue from human Ulcerative Colitis (UC) patients Colonic biopsies were collected from healthy individuals (Normal) and Ulcerative Colitis (UC) patients. qRT-PCR was used to measure levels of mature miR-146a RNA within the collected tissue, and expression was normalized to 5S rRNA. *n* = 10 (normal), *n* = 35 (UC).

Because we found a role for miR-146a in regulating colitis in mice, ulcerative colitis (UC) patients were examined to determine if miR-146a levels correlate with intestinal disease in humans. We obtained ulcer-free colon biopsies from non-diseased individuals (normal) and UC patients. Mature miR-146a RNA levels were measured, and we found that miR-146a expression was significantly elevated in UC patients compared to healthy controls (Figure 6). These data further demonstrate correlation of miR-146a expression in human UC and CD, in which previous studies have found associations of miR-146a expression and polymorphisms with IBD type and risk [24, 66–68]. Our results indicate that higher miR-146a levels correlate with UC, and further investigation may distinguish the severity of disease and/or development of colorectal cancer (CRC) as a result of UC.

## DISCUSSION

Our study provides the first genetic evidence that miR-146a, a microRNA that is important in a variety of immunological contexts, is also relevant within intestinal tissues. In previous studies, miR-146a was shown to be an anti-inflammatory miRNA by repressing genes that promote pro-inflammatory signaling. In this way, loss of miR-146a results in worsened disease susceptibility and severity in most model systems [23, 27, 29, 33, 69]. Our study is unique in that loss of miR-146a resulted in a more beneficial host outcome following DSS-induced colitis. This appears to be the result of miR-146a down- regulating expression of genes and cell types that fortify the intestinal barrier.

Previous work has shown that miR-146a down regulates pro-inflammatory pathways, including signaling downstream of the TLR/IL1R adaptor protein MyD88 in addition to IFN activated Stat1, in a variety of peripheral tissues [27, 31, 56]. Pathway analysis of our gene expression data set indicates that miR-146a represses these same pathways in the gut. MyD88 signaling is actually beneficial in the intestines, as MyD88−/− mice develop worsened DSS colitis [16–18, 54]. One mechanism used by TLR/MyD88 signaling to protect the gut is induction of antimicrobial peptides such as RegIIIγ [40], mucins [70], IL-18 [52], and other molecules that are produced by intestinal epithelial cells. Consistent with miR-146a repressing this pathway, miR-146a−/− mice are more protected from DSS colitis compared to control mice, and all of these TLR-MyD88 induced, host protective genes are elevated in the small intestines of miR-146a−/− mice. In addition to TLR/MyD88 signaling, we also see predicted de-repression of the IFN activated factor Stat1, a pathway that has also been shown to protect gut tissues. Taken together, miR-146a−/− mice appear to be protected from DSS colitis through a mechanism involving enhanced TLR/MyD88 and IFN/Stat1 signaling. However, the additional miR-146a targets identified in our RNA-Seq experiment, as well as ICOS function in Tfh cells, also appear to be involved.

We also observed enhanced GC responses in the PPs of miR-146a−/− mice, including increases in Tfh and GC B cells and elevated IgA levels in the intestinal lumen. This correlated with increased ICOS expression on T cells, presumably through direct miR-146a targeting of ICOS mRNA and other Tfh-related genes [32]. Interestingly, we have recently reported that T cell specific MyD88 signaling is critical for Tfh cell formation and downstream formation of GC B cells and IgA production [55]. Further, this pathway impacted the host microbiota through selective IgA binding to commensal populations, and this was beneficial to host intestinal health. It has been shown that miR-146a negatively regulates downstream TLR signaling pathways (e.g. TRAF6) in CD4^+^ T cells, and our data suggest that miR-146a acts as a negative regulator of T cell intrinsic TLR signaling that triggers increased GC responses, luminal IgA and intestinal health. Therefore, this appears to be an additional mechanism underlying the miR-146a−/− mouse gut phenotype.

Intestinal miR-146a expression is enriched in cells of the hematopoietic lineage, although this does not rule out the possibility that the observed low-level expression of this miRNA in CD45^−^ cells may also be functional. Even so, we observed protection from colitis in WT mice upon bone marrow reconstitution with miR-146a−/− cells, indicating that it plays a required role in the hematopoietic compartment. Future work with miR-146a conditional knockout mice will allow us to determine the relative contribution of distinct cell types to the miR-146a intestinal phenotype, including an assessment of T cell and IEC intrinsic roles for this miRNA.

We have found that WT and miR-146a−/− mice have different gut microbial populations, demonstrating that miR-146a plays some role in shaping commensal microbiota. Although the biological relevance of the altered populations is currently unclear, and does not appear to impact the DSS phenotype, these altered microbial populations may be playing important roles in other intestinal conditions such as Crohn's Disease and CRC. miR-146a reshaping of the microbiota might also be relevant in extra-intestinal disorders [71], such as obesity [72] and diabetes [73, 74], multiple sclerosis [75], and infections [76–78], as both miR-146a and the microbiota have been implicated in these situations.

While miR-146a−/− mice are protected from DSS colitis, a model of intestinal barrier function, the functional role of miR-146a during CRC is unknown. In fact, miR-146a is thought of as a tumor suppressor in many cancer settings [27]. In addition, miR-146a has also been shown to play a role in protection from ischemia/reperfusion injury [30] and may contribute to early microbial colonization in the gut [34]. Thus, the benefits of miR-146a function within the gut may outweigh its adverse effects during colitis. This might explain why miR-146a has evolved to play a negative regulatory role during at least some forms of colitis.

Our study shows that miR-146a has clinical relevance within the intestine, as UC patients on average show elevated levels of this miRNA within the colon tissue. Thus, targeting miR-146a therapeutically might prove to be an effective treatment of this condition. However, one must keep in mind that using miR-146a as a therapy to treat extra-intestinal disorders may exacerbate intestinal disease. Future studies need to be done to examine the interplay of miR-146a expression within different contexts in human patients to determine how to design targeted delivery of miR-146a therapeutics. It is also possible that inhibition of miR-146a may be beneficial for some human patients with intestinal disease, but again, proper targeting to gut cells will be important

## MATERIALS AND METHODS

### Animals

All WT and miR-146a−/− mice are on a C57BL/6 background and were bred and housed in a specific pathogen-free mouse facility at the University of Utah, USA. WT Germ-free mice were housed in the germ-free mouse facility at the University of Utah, USA. All mice were 6-8 weeks old at time of experimentation. Experimental procedures were performed with the approval of the Institutional Animal Care and Use Committee (IACUC) of University of Utah, USA.

### CD45 cell sorting

WT and miR-146a−/− colons and small intestines were obtained, mesenterium was removed, and the tissues were rinsed in PBS. Intestines were cut longitudinally, cleaned of mucus and feces, and cut into fragments. Fragments were then placed in a cell dissociation solution made of calcium-free HBSS with 5mM EDTA and 10uM HEPES (colon) or 1 mM DTT (small intestine) and incubated for 45 minutes at 37°C. Following cell dissociation, samples were strained into 100uM filters and flow-through was collected and placed on ice. Intestinal fragments were then placed in a digestion mix containing calcium-free HBSS, 5% FBS, 1 U/mL Dispase (Roche), 0.5 mg/mL Collagenase D (Roche), and 25 ug/mL DNase I (Worthington) and incubated at 37°C for 45 minutes. Tissue was strained through a 40uM strainer and flowthrough was collected. Cell suspensions from the cell dissociation and digestion steps were combined and resuspended in flow cytometry buffer (HBSS, 10mM HEPES, 2mM EDTA, 0.5% w/v FBS). Cells were stained with CD45-FITC antibody (Biolegend) and 7-AAD viability staining solution (Biolegend). Fluorescence-activated cell sorting was performed using the FACS Aria (BD Biosciences) gating on singlet, live (7-AAD^−^) cells and sorting CD45^+^ from CD45^−^ cells. Qiazol (Qiagen) was added to WT and miR-146a−/− small intestine and colon samples containing > 10,000 CD45^+^ or CD45^−^ sorted cells. RNA was extracted from sorted cells using the miRNeasy kit (Qiagen) and miR-146a expression was quantified via qPCR as described above.

### Gene expression analysis

1 cm pieces of distal colon and ileum of the small intestine (SI) were removed, gently cleaned to remove mucus and feces, and rinsed in PBS. The colon and SI pieces were then placed in Qiazol (Qiagen). Tissues were homogenized and RNA was extracted using the RNeasy or miRNeasy spin column kit (Qiagen) and quality was measured. Samples were prepared and sequenced at the Microarray and Genomic Analysis Core Facility at University of Utah, USA. Libraries were prepared using RiboZero treatment and sequencing was done with Illumina TruSeq Stranded RNA-seq in 50 single-end cycles. Sequences were aligned and annotated with help of the Bioinformatics Core Facility at University of Utah, USA. Pathways were examined by uploading analyzed data into the Ingenuity IPA software program and performing a core analysis on genes upregulated or downregulated by 2-fold or more, (with Phred-transformed FDR > 10).

Quantitative real-time PCR (qRT-PCR) was performed using RNA prepared in the manner described above, which underwent randomly primed cDNA synthesis using qScript cDNA SuperMix (Quanta Biosciences). qRT-PCR was performed using SYBR green detection with GoTaq qPCR master mix (Promega) or LightCycler 480 SYBR Green I Master (Roche). All primers were purchased from Integrated DNA Technologies (IDT) and signals were normalized to L32. Primers sequences are listed in Table 1.

**Table 1:** Primer sequences

Gene	Forward primer (5′-3′)	Reverse primer (5′-3′)
Reg3b	CTGCCTTAGACCGTGCTTTC	ATAGGGCAACTTCACCTCAC
Reg3g	TTCCTGTCCTCCATGATCAAA	CATCCACCTCTGTTGGGTTCA
Ceacam1S	CTGGCATCGTGATTGGAGTT	CAGAAGGAGCCAGATCCG
Ceacam1L	GCGAGATCTCACAGAGCACA	GCTGGGAATTGAAGTTCAGG
Il18	GCCTCAAACCTTCCAAATCA	TGGATCCATTTCCTCAAAGG
Nos2	CAGCTGGGCTGTACAAACCTT	CATTGGAAGTGAAGCGGTTCG
Oasl1	CCAGGAAGAAGCCAAGCACCATC	AGGTTACTGAGCCCAAGGTCCATC
Ido1	GGCTTTGCTCTACCACATCCAC	TAGCCACAAGGACCCAGGG
L32	AAGCGAAACTGGCGGAAAC	TAACCGATGTTGGGCATCAG

Mature miR-146a was quantified using the miRCURY LNA Universal RT microRNA PCR cDNA synthesis and SYBR Green Master Mix kits (Exiqon). microRNA levels were normalized to 5S rRNA (primer from Exiqon) and the LNA miR-146a primer was purchased from Exiqon.

### Lamina propria and Peyer's patch isolation/flow cytometric analysis

WT and miR-146a−/− colons and small intestines were obtained; the entire colon was cut from the anus to the cecum and the first 20 cm of the small intestine beginning with the ileum was used. Mesenterium, connective tissue, and Peyer's Patches were removed; the tissues were rinsed in PBS. Intestines were cut longitudinally and mucus and feces were removed. Tissues were then cut into fragments and were then placed in a cell dissociation solution made of calcium-free HBSS (Corning) with 5mM EDTA and 10mM HEPES (colon) or HBSS with 5mM EDTA and 1 mM DTT (small intestine) and incubated for 45 minutes at 37°C. Following cell dissociation, samples were strained into 100uM filters and flow-through was discarded. Intestinal fragments were then placed in a digestion mix containing calcium-free HBSS with 5% w/v FBS, 1 U/mL Dispase (Roche), 0.5 mg/mL Collagenase D (Roche), and 25 ug/mL DNase I (Worthington) and incubated at 37°C for 45 minutes. Tissue was strained through a 40uM strainer and flowthrough containing LP cells was collected. Cells were subjected to a 40%-80% Percoll gradient spin, then washed and resuspended in FACS buffer (HBSS, 10mM HEPES, 2mM EDTA, 0.5% w/v FBS). Cells were restimulated with PMA (Sigma), ionomycin (Sigma), and Golgi plug (BD) for 4 hours, were surface stained with fluorophore-conjugated antibodies B220-FITC (Biolegend), CD11b-APC (Biolegend), CD3e-PerCP Cy5.5 (Biolegend), and CD4-FITC (eBioscience) before they were fixed and permeablized overnight. Cells were washed and intracellulary stained with FoxP3-APC (eBioscience), IL-10-PE (eBioscience), IFNγ-PE (eBioscience), and IL-17a-APC (eBioscience) before washing and running in a flow cytometer (LSR Fortessa, BD Biosciences).

Peyer's Patches were removed from the small intestines of WT and miR-146a−/− mice. They were gently passed through a 40 μM filter in PBS in order to obtain white blood cells. Cell suspensions were then washed and resuspended in flow buffer (HBSS, 10mM HEPES, 2mM EDTA, 0.5% w/v FBS). Germinal center B cells were identified by staining with antibodies against GL7 (ebioscience), FAS (BD Pharmagen), IgD (Biolegend) and B220 (Biolegend). The Tfh cells were identified by staining with antibodies against CD3ε (eBioscience), CD4 (eBioscience), CXCR5 (ebioscience), PD-1 (Biolegend), and/or ICOS (Biolegend), and gating based on isotype or unstained controls.

### Microbiota analysis

WT and miR-146a−/− mice were sacrificed; their colons were collected and cut open longitudinally. Feces was removed and flash-frozen in liquid nitrogen. Microbial DNA was extracted from feces using the PowerSoil DNA Isolation kit (MoBio). From the extracted DNA, 16S rDNA V4 region PCR amplicons were sequenced on the MiSeq platform (Illumina) using the 2×250bp protocol yielding pair-end reads with a mean merged length of ∼247 bps [79]. Following sequencing, raw BCL files were retrieved from the MiSeq platform and called into fastqs by Casava v1.8.3 (Illumina). The read pairs were merged using USEARCH v7.0.1090 with the fastq_mergepairs parameters ‘-fastq_minovlen 20 -fastq_truncqual 5 -fastq_maxdiffs 5 -fastq_maxmergelen 350 -minhsp 8′, then filtered with fastq_filter to exclude sequences with more than 0.5 expected errors over the length of the merged read. Bowtie2 v2.2.1 was used to identify and remove reads mapping to the PhiX genome. Sequences were next demultiplexed based on exact barcode matches and then clustered into 97% identity operational taxonomic units (OTUs) using the UPARSE pipeline [80]. Phylogenetic annotation of OTUs was achieved by mapping the UPARSE OTUs to the SILVA v111 database with a minimum identity of 97% [81]. The resulting OTU table representing 2,301,977 sequences in 582 OTUs was rarefied to an even depth of 21,707 reads per sample prior to calculation of alpha-diversity, beta-diversity, taxonomic summaries, and related analyses in QIIME and the phyloseq R package [82–87]. Significance of categorical variables were determined using the non-parametric Kruskal-Wallis test. Correlation between two continuous variables was determined with linear regression models, where p-values indicate the probability that the slope of the regression line is zero. Principal coordinate plots employed the Monte Carlo permutation test to estimate p-values. All p-values were adjusted for multiple comparisons with the FDR algorithm.

### Colitis

Dextran sodium sulfate (DSS) colitis was induced by dissolving DSS (MP Biomedicals, 36,000-50,000 mw) in autoclaved water at a 3.5% w/v concentration. DSS water was placed in cages of WT and miR-146a−/− mice, where they drank DSS water *ad libitum* for 8 days; water was changed to fresh DSS water on day 4. Mice were weighed daily and percent weight loss was tracked over time. Mice were sacrificed and analyzed for disease severity on day 8, where colon length was measured, and colons were fixed in formalin for histology. For qRT-PCR of DSS-treated colon segments, colon tissue was subjected to a LiCl purification following RNA isolation to remove residual DSS [88].

### Microbiota transfers

Donor microbiota was obtained by scraping mucus and feces from colons of WT and miR-146a−/− mice. Scrapings were placed in sterile PBS, centrifuged at 400G to remove debris, then resuspended and centrifuged at 8000G to obtain bacteria. Samples were resuspended in sterile PBS and flash frozen for gavage. Recipient mice were treated with an antibiotic cocktail of Gentamycin (Goldbio), Ampicillin (Cellgro), Neomycin Sulfate (Fisher), and Erythromycin (Fisher); each at 0.5g/L in drinking water for 7 days. After 7 days, drinking water was changed to regular water and 100ul of donor WT or miR-146a−/− bacteria was orally gavaged into recipient mice every day for 7 days. Following gavage, mice rested for 7 days to allow for microbiota stabilization. Finally, DSS colitis was administered in recipient mice using the methods described above.

### miR-146a expression in human patients

Colon biopsies were obtained from normal (healthy) or Ulcerative Colitis patients. Frozen biopsy samples were processed in RNAlater-ICE transition solution overnight at −20°C and homogenized using Qiagen TissueRupter. Total RNA was extracted from the resultant tissue suspension using TRIzol LS reagent as per the manufacturer's instructions. qPCR was performed to assay expression levels of mature miR-146a using the methods described above.

### Statistics

A Student's t-test was utilized to determine significant p-values when comparing two groups, unless otherwise noted. †≤0.12; * p ≤0.05; ** p≤0.01; *** p≤0.001; **** p≤0.0001. For p-values during DSS colitis, individual t-tests were performed at each time point. Outliers from experiments were removed using the Graphpad Prism 6 “identify outliers” statistical function. See “microbiota analysis” and “gene expression analysis” sections for explanation of statistics in those experiments.

## SUPPLEMENTARY MATERIAL FIGURES


