# Supplementary material for: MicroRNA-146a constrains multiple parameters of intestinal immunity and increases susceptibility to DSS colitis
Source: Oncotarget. 2015 Sep 10;6(30):28556–72. doi: 10.18632/oncotarget.5597 (PMC4745677; doi:10.18632/oncotarget.5597)
Supplement: Supplementary file 1 [file oncotarget-06-28556-s001.pdf]

MicroRNA-146a constrains multiple parameters of intestinal immunity and increases susceptibility to DSS colitis

Supplementary Material

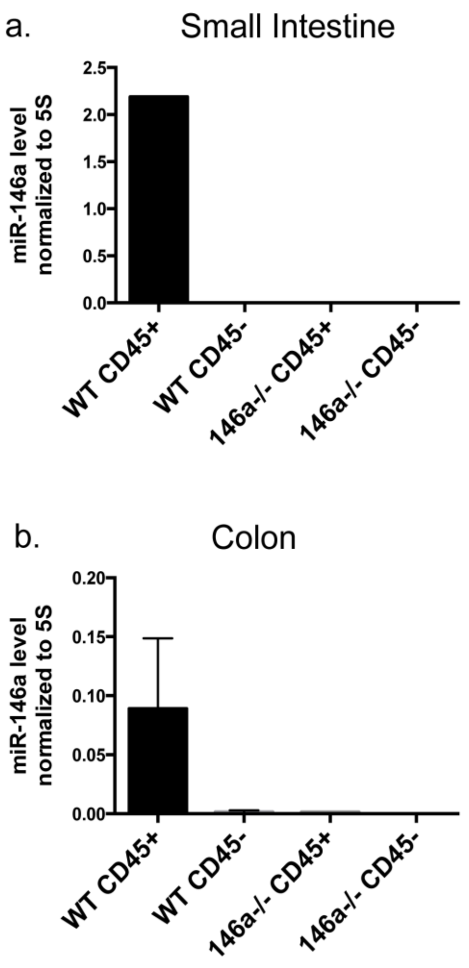

Supplementary Figure 1. miR-146a is enriched in $CD45^+$ cells of the colon and small intestine. Cells were dissociated from WT and miR-146a-/- small intestines and colons, stained for viability and CD45, and $CD45^+$ and $CD45^-$ cells were sorted via FACS. RNA was extracted from sorted cells, and qRT-PCR was run to measure mature miR-146a expression. miR-146a expression in $CD45^+$ and $CD45^-$ cells of the small intestine **(a)** and colon **(b)**. n=3 (a and b)

a. Significant gene expression changes in small intestine (>2-fold change, FDR>10)

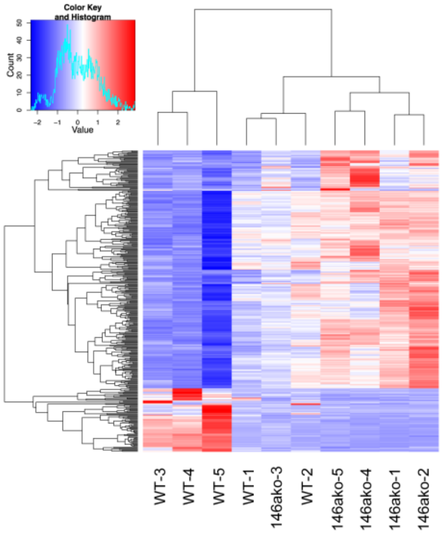

b. Significant gene expression changes in colon – miR-146a-/- over WT (FDR>10)

Gene name	Fold Change
Selenbp2	5.749662668
Tpm3-rs7	3.189507706
Edn1	2.377290752
Klhl24	2.061948947
Pbsn	-2.114540161
Ighv6-6	-2.168731855
Fam115e	-2.168880239
Neat1	-2.171490443
5830417I10Rik	-2.22453276
Prune2	-2.225547451
Tpm3	-2.292656429
Tgm4	-2.420075924
Sprr2a1	-2.546951018
Dab1	-2.57060169
Slc26a2	-2.67142886
9530053A07Rik	-2.871575472
Gm23935	-3.845753444
NA	-6.825406786

Supplementary Figure 2. miR-146a impacts global gene expression within the small intestine and regulates few colonic genes. RNA-seq was performed on ileal tissue from WT and miR-146a-/- mice. Heat map indicates a fold change in expression of genes with a >2-fold change when comparing miR-146a-/- mice with WT, p<0.05, Phred-transformed FDR>10 **(a)**. RNA-seq was performed on colonic tissue from WT and miR-146a-/- mice. Table indicates fold change in expression of genes with >2-fold change, comparing miR-146a-/- mice with WT, Phred-transformed FDR>10 **(b)**.

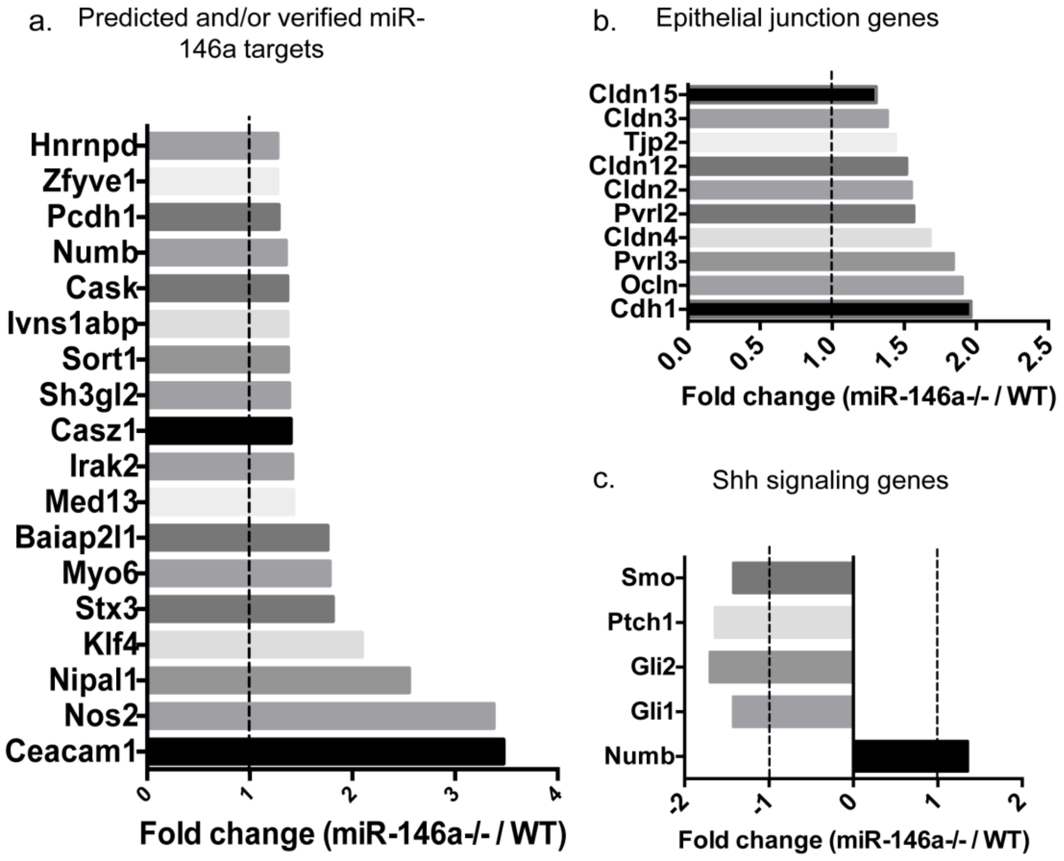

Supplementary Figure 3. miR-146a regulates the expression of epithelial junctions and predicted targets within the small intestine. Expression levels of selected predicted and/or experimentally verified mmu-miR-146a targets (according to Targetscan and miRTaRBase), comparing miR-146a-/- with WT small intestine. Targets genes shown had >1.3-fold expression change and Phred-transformed FDR>5 **(a)**. Expression levels of select intestinal epithelial junction genes in the small intestine when comparing miR-146a-/- to WT **(b)**. Expression levels of miR-146a target NUMB and downstream SHH signaling genes in the small intestine when comparing miR-146a-/- to WT **(c)**.

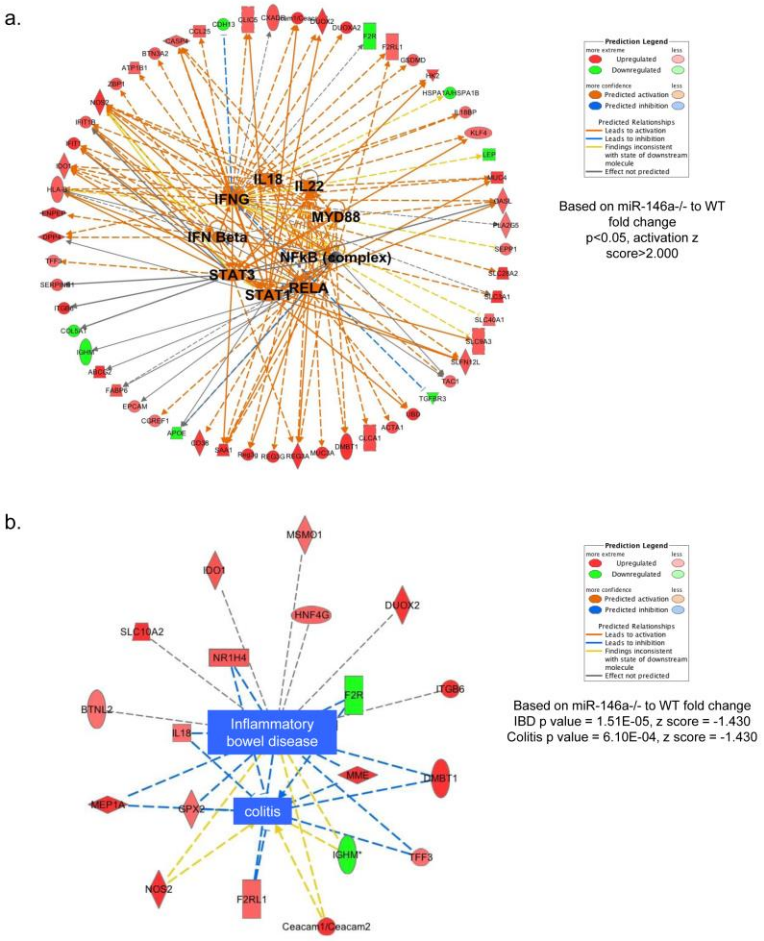

Supplementary Figure 4. IPA predictions indicate that miR-146a downregulates proinflammatory pathways in the small intestines and will enhance IBD. Ingenuity Pathway Analysis (IPA) showing predicted activation of proinflammatory pathways MyD88, NFkB, Stat1, Stat3, IFNb, IFNg, IL-22, and IL-18 within miR-146a-/- small intestines mice compared to WT; based on genes that are upregulated in RNA-seq data of ileal tissues (found in outside wheel of red and green shapes) **(a)**. Ingenuity Pathway Analysis (IPA) showing predicted inhibition of inflammatory bowel disease and colitis in miR-146a-/- mice, based on genes that are upregulated as measured by RNA-seq of the ileum **(b)**.

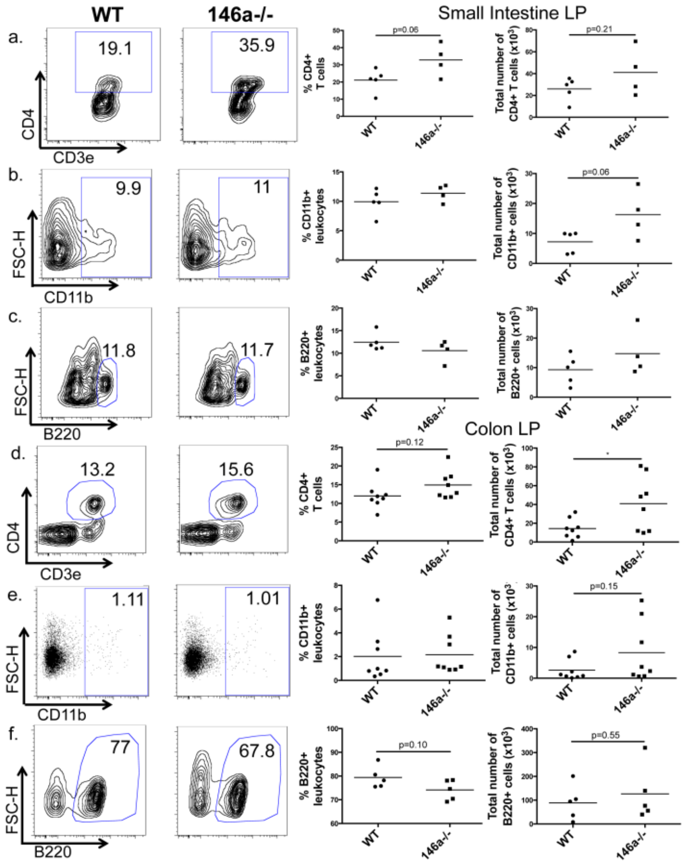

Supplementary Figure 5. miR-146a-/- mice have altered $CD4^+$ T cell populations within the intestinal lamina propria. Lymphocytes were isolated from the small intestinal lamina propria, and flow cytometry was utilized to examine immune cell populations within this tissue. Displayed are representative flow plots, percentages, and total numbers of $CD4^+$ $CD3e^+$ T cells within the small intestinal LP. All populations were first gated on lymphocytes using the FSC/SSC gate, followed by the CD4 and CD3e gating shown **(a)**. Representative flow plots, percentages, and total numbers of $CD11b^+$ cells within the small intestinal LP. All populations were first gated on lymphocytes using the FSC/SSC gate, followed by the CD11b gating shown **(b)**. Representative flow plots, percentages, and total numbers of $B220^+$ cells within the small intestinal LP. All populations were first gated on lymphocytes using the FSC/SSC gate, followed by the

B220 gating shown **(c)**. Displayed are representative flow plots, percentages, and total numbers of $CD4^+$ $CD3e^+$ T cells within the colonic LP. All populations were first gated on lymphocytes using the FSC/SSC gate, followed by the CD4 and CD3e gating shown **(d)**. Representative flow plots, percentages, and total numbers of $CD11b^+$ cells within the colonic LP. All populations were first gated on lymphocytes using the FSC/SSC gate, followed by the CD11b gating shown **(e)**. Representative flow plots, percentages, and total numbers of $B220^+$ cells within the colonic LP. All populations were first gated on lymphocytes using the FSC/SSC gate, followed by the B220 gating shown **(f)**. n=5 (WT) and 4 (146a-/-) (a-c), n=8 (d-f).

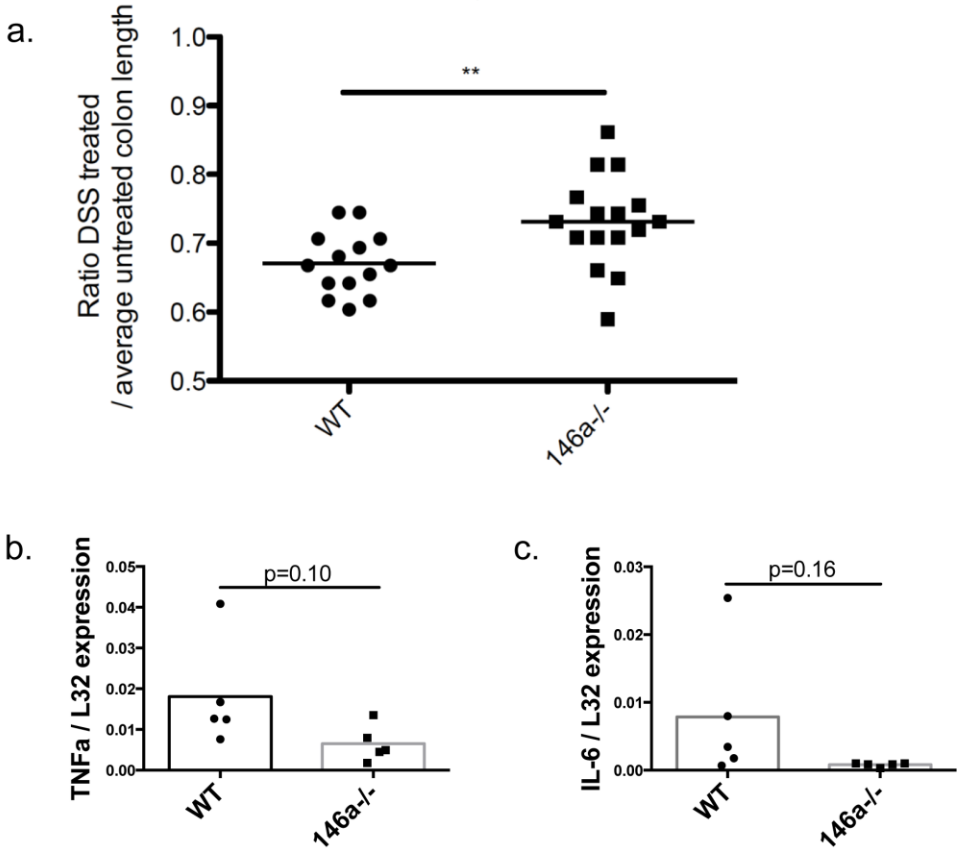

Supplementary Figure 6. DSS treated miR-146a-/- have decreased colon shortening and expression of inflammatory genes. Colon shortening in WT and miR-146a-/- mice following 3.5% DSS treatment, as calculated by DSS-treated colon length divided by average untreated colon length for each respective genotype **(a)**. Expression of TNFα **(b)** and IL-6 **(c)** in WT and miR-146a-/- DSS-treated colons, as measured by qRT-PCR. n=13 (WT) and 15 (146a-/-) (a), n=5 (b and c).

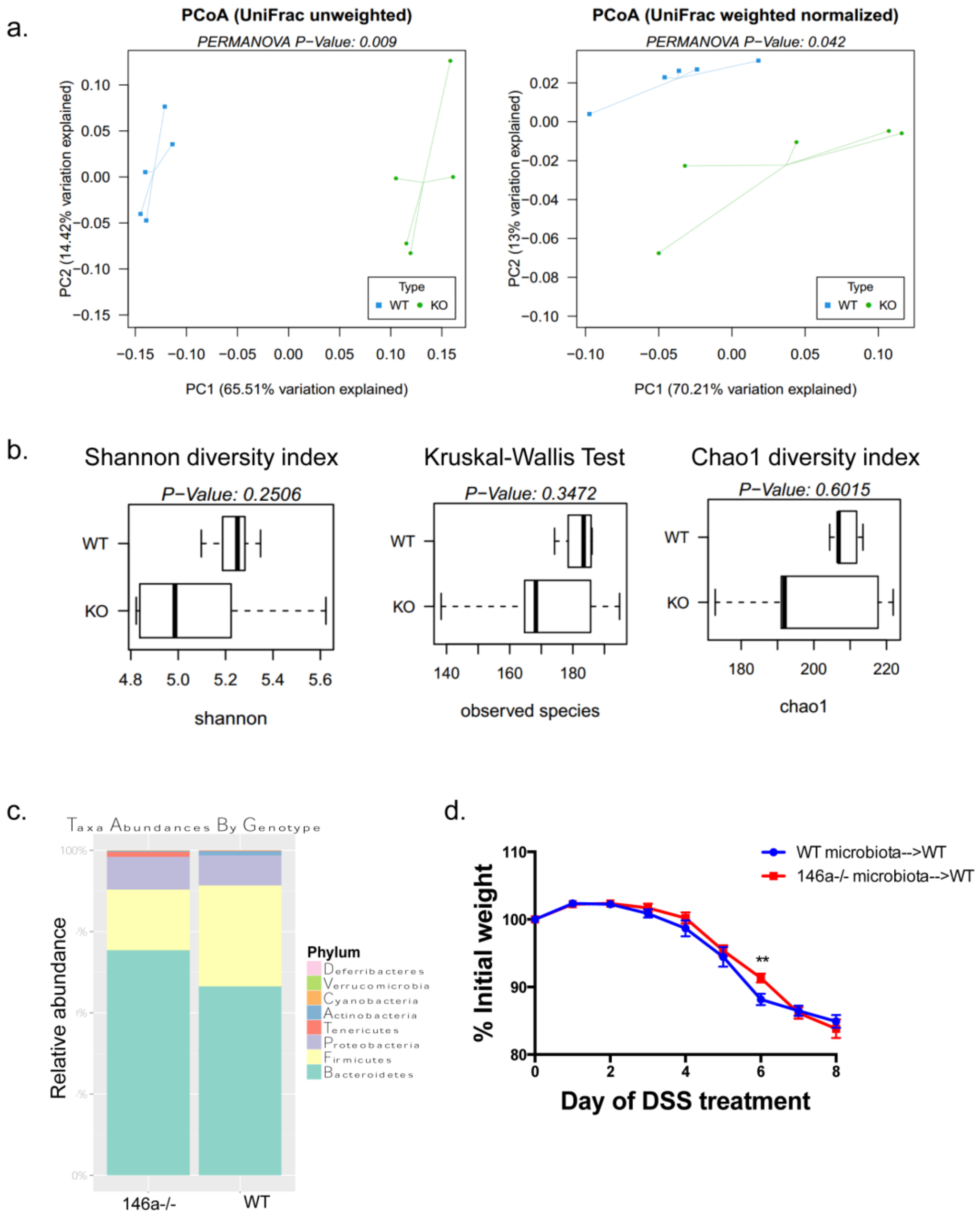

Supplementary Figure 7. miR-146a affects beta diversity of fecal microbiota, but not alpha diversity or phyla ratios. Feces was collected from WT and miR-146a-/- mice; total bacterial 16s rDNA was extracted and next-generation sequencing was performed to measure microbiota populations. Principle coordinate analysis plots of fecal microbiota comparing WT communities (blue) with miR-146a-/- (KO) communities (green),

measured via 16s rDNA sequencing; using unweighted Unifrac distance and the weighted, normalized Unifrac distance measure **(a)**. Alpha diversity measures of WT and miR-146a-/- (KO) fecal microbiota communities, using the Shannon diversity, Kruskal-Wallis, and Chao1 diversity indices **(b)**. Relative abundances of 8 major phyla within the kingdom Bacteria, comparing miR-146a-/- and WT fecal communities. No statistical differences in relative abundance were found at the phyla level. WT and miR-146a-/- mice were housed separately within a specific pathogen-free (SPF) mouse facility; n=5 **(c)**. WT or miR-146a-/- intestinal microbiota were transferred to antibiotic-treated WT mice via oral gavage, and recipient mice were administered 3.5% DSS. Weight change of WT and miR-146a-/- microbiota recipient mice during DSS colitis, as measured by percent initial weight **(d)**. n=19 (WT) and 18 (146a-/-); 3 independent experiments.
